# A Comparison of Synonymous Codon Usage Bias Patterns in DNA and RNA Virus Genomes: Quantifying the Relative Importance of Mutational Pressure and Natural Selection

**DOI:** 10.1155/2013/406342

**Published:** 2013-10-02

**Authors:** Youhua Chen

**Affiliations:** Department of Zoology, University of British Columbia, Vancouver, BC, Canada V6T 1Z4

## Abstract

Codon usage bias patterns have been broadly explored for many viruses. However, the relative importance of mutation pressure and natural selection is still under debate. In the present study, I tried to resolve controversial issues on determining the principal factors of codon usage patterns for DNA and RNA viruses, respectively, by examining over 38000 ORFs. By utilizing variation partitioning technique, the results showed that 27% and 21% of total variation could be attributed to mutational pressure, while 5% and 6% of total variation could be explained by natural selection for DNA and RNA viruses, respectively, in codon usage patterns. Furthermore, the combined effect of mutational pressure and natural selection on influencing codon usage patterns of viruses is substantial (explaining 10% and 8% of total variation of codon usage patterns). With respect to GC variation, GC content is always negatively and significantly correlated with aromaticity. Interestingly, the signs for the significant correlations between GC, gene lengths, and hydrophobicity are completely opposite between DNA and RNA viruses, being positive for DNA viruses while being negative for RNA viruses. At last, GC12 versus G3s plot suggests that natural selection is more important than mutational pressure on influencing the GC content in the first and second codon positions.

## 1. Introduction

Codon usage is not a random event [1]. Codon usage bias has been broadly observed, and different mechanisms have been proposed to explain the bias patterns, for example, mutation pressure, translational efficiency, gene length [2], dinulcoetide bias [3], tRNA abundance [4], organ specificity [5], and so on. Codon usage bias patterns have been broadly studied in recent years, especially for virus genomes [3, 6, 7]. However, most of these previous studies only consider a specific virus or a specific virus clade [8–10], a global comparison of virus codon usage bias patterns is still largely lacking, even though some literature had worked on many RNA and DNA viruses as whole [11–13]. A holistic observation and comparison of codon usage patterns over different clades of viruses would throw new insights into virus genome explicitly. To cope with such a knowledge gap, in the present study, I analyzed codon usage patterns for the available 2317 virus genomes for the purpose of providing a more robust and integrated understanding of synonymous codon usage patterns. 

 Given the accumulation of genome sequences from different viruses in GenBank database, another purpose of the present study is to quantify the relative contribution of mutation pressure and natural selection on influencing codon usage patterns of virus genomes. I could achieve such an objective by introducing a new statistical method called variance partitioning to quantitatively examine the separated role of different mechanisms on synonymous codon usage patterns of viruses.

## 2. Materials and Methods

### 2.1. Sequence Data

The complete genome sequences for 2317 different virus species were originally obtained from GenBank database (http://www.ncbi.nlm.nih.gov/genomes/VIRUSES/viruses .html). Because some viruses have been sequenced for multiple times using different strains, for avoiding sampling bias, only one from these multiple genomic sequences for the same virus is used. Furthermore, because RNA and DNA viruses are very different on their codon usage biase patterns [12], RNA and DNA viruses are analyzed separately. Genomes belonging to other types of viruses, such as Retro-transcribing viruses, are not considered. 

Consequently, 786 DNA viruses and 725 RNA viruses are retained for all subsequent analyses, representing around 65% of the total virus species in the NCBI Genome database. By extracting all the valid open reading frames (ORFs) from each genome sequence and removing problematic ones (including short-length (less than 350 bp) ORFs, overlapping ORFs for different genes/transcripts, ORFs with nontranslatable codons, and ORFs without synonymous codons), 35818 ORFs for DNA viruses and 2743 ORFs for RNA viruses are kept for calculating codon usage indices and performing multivariate analyses. 

### 2.2. Measures of Relative Synonymous Codon Usage (RSCU)

Relative synonymous codon usage values of each codon in a gene are calculated to investigate the characteristics of synonymous codon usage. The RSCU index is calculated as follows [14]:

(1) RSCU=gij×nj∑injgij,

where *g*_*ij*_ is the observed number of the *i*th codon for the *j*th amino acid which has *n*_*i*_ kinds of synonymous codons. Codons with higher (or lower) selected frequencies have higher (or lower) RSCU values. When the corresponding RSCU values of a codon are close to 1, it is used randomly and evenly. 

### 2.3. Effective Number of Codons

The effective number of codons (ENC) is a measure of bias from equal codon usage in a gene [15]. The calculation formula is

(2) ENC=2+9F−2+1F−3+5F−4+3F−6,

where ${\overset{-}{F}}_{k}\;(k=2,3,4,6)$ is the mean of *F*_*k*_ values for the *k*-fold degenerate amino acids, which is estimated using the formula as follows:

(3) Fk=nS−1n−1,

where *n* is the total number of occurrences of the codons for that amino acid and

(4) S=∑i=1k(nin)2,

where *n*_*i*_ is the total number of occurrences of the *i*th codon for that amino acid. 

*N*_*c*_ ranges from 20 for the strongest bias (where only one codon is used for each amino acid) to 61 for no bias (where all synonymous codons are used equally).

For elucidating the relationship between GC3s and ENC values, the expected ENC values for different GC3s are calculated as follows:

(5) ENCexpected=2+s+29s2+(1−s)2,

where *s* denotes the value of GC3s [6]. The observed and expected ENC values are compared to determine the influence of nucleotide compositional constraint on structuring synonymous codon usage bias. 

### 2.4. Codon Adaptation Index

The codon adaptation index (CAI) estimates the extent of bias toward codons that are known to be favored in highly expressed genes [16]. In the present study, for simplicity, the *Escherichia coli* optimal codons are used as the reference. 

### 2.5. Indices for Measuring Chemical Properties of Amino Acids

 Hydrophobicity (GRAVY) and aromaticity (AROMO) of conceptually translated gene product may be factors influencing codon usage bias patterns [17]. As such, I quantify both indices to reveal the evidence of natural selection on codon usage bias.

 For hydrophobicity index [17], it is calculated as

(6) GRAVY=1N∑i=1Nki,

where *N* is the number of amino acids and *k*_*i*_ is the hydrophobic index of the *i*th amino acid.

 For aromaticity index [17], it is calculated as

(7) AROMO=1N∑i=1Nvi,

where *v*_*i*_ is either 1 (for an aromatic amino acid) or 0 (for a nonaromatic amino acid) and *N* is the number of amino acids.

### 2.6. Correspondence Analysis and Canonical Correspondence Analysis

 In addition to utilizing conventional correspondence analysis (CA) [17], in the present study, I introduce a new method, namely, canonical correspondence analysis (CCA) [18], which could help reveal the principal trends of codon usage bias patterns and identify the most correlated variables simultaneously. CCA method has been broadly applied in ecological studies [18, 19]. However, it might be the first time to be applied to study synonymous codon usage patterns for viruses in the present study. 

The mathematical formulation for CCA method [18, 19] is a bit complicated in comparison to its linear analogue redundancy analysis (RDA) [19, 20] because it requires data transformation. As such, the calculation steps for RDA are present here for demonstrating the calculation core steps of CCA.

 Assuming that one has the codon usage matrix *Y* and the matrix of explanatory variables (codon usage indices) *X* (both have the same rows), then the RDA procedure is to predict the elements (codon usage values) in the matrix *Y* as

(8) Y^=X[XTX]−1XTY,

where the subscript *T* denotes the transpose of the matrix and −1 denotes the inverse of the matrix. 

Thus the covariance matrix for the predicted codon usage matrix $\hat{Y}$ is (*n* denotes the row number)

(9) M=1n−1Y^TY^.

The RDA or CCA method is to decompose the above matrix *M* into normalized eigenvalues *E* and normalized eigenvector matrix *U*. Elements from *E* ranked from high to low represent the explained proportion of total variation in the codon usage patterns, while the corresponding eigenvectors *U* can be used to obtain sample scores and biplots when generating the 2-dimensional plots.

### 2.7. Quantifying the Influence of Mutation Pressure and Selection Pressure Using Variation Partitioning

 Variation partitioning is a relatively new method for helping elucidate the influence of each group of explanatory variables in multivariate statistics [21]. Variation partitioning has been broadly applied in ecological and evolutionary studies [19]. For quantifying the influence of mutation selection, I consider the metrics related to codon contents, like GC, GC3s, A3s, T3s, C3s, and G3s contents, as the factors reflecting mutational pressure. In contrast, the indices CAI, all kinds of protein properties, including hydrophobicity and aromaticity, are regarded as the representative of natural selection [3, 17, 22]. For simplicity, the mathematical formulation for variation partitioning technique is as follows [21, 23, 24].

 Supposing that there are two groups of explanatory variables in two matrices *X*_1_ and *X*_2_, the total variation *S* in the codon usage table matrix *Y* with *n* rows is written as

(10) S=1(n−1)Trace((Y−Y−)T(Y−Y−)),

where a hyphen above the variable(s) denotes the mean(s).

 Then the proportion of variation *R*_1_ only explained by the group of explanatory variables *X*_1_ is obtained as

(11) Y1=X2[X2TX2]−1X2TY,Y1res=Y−Y1,X1res=X1−X2[X2TX2]−1X2TX1,Y^1=X1res[X1res TX1res]−1X1res TY1res,R1=Trace((1/(n−1))(Y^1−Y−1)T(Y^1−Y−1))  S.

The percentage of total variation *R*_2_ attributed to the explanatory variable group *X*_2_ is calculated following the same procedure as above.

Finally, the percentage of total variation explained by the interaction of the two variable groups *X*_1_ and *X*_2_ requires the determination of the percentage of variation (*R*_12_) explained by all the variables *X*, the matrix of which combines matrices *X*_1_ and *X*_2_ together:

(12) Y12=X[XTX]−1XTY,R12=Trace((1/(n−1))(Y12−Y−12)T(Y12−Y−12))S.

Thus, the proportion of variation that cannot be explained by any current explanatory variables is determined by

(13) R0=1−R12.

Then, the percentage of total variation explained by the interaction of the two variable groups is given by,

(14) R1∩2=R12−R1−R2.

In a summary, *R*_1_, *R*_2_, *R*_1∩2_ and *R*_0_ are the focused explained variation for the present study. 

### 2.8. Statistical Programs

 Multivariate analyses, including CA, CCA, and variation partitioning methods, were implemented in *R* [25] package “*vegan*” [26]. All other codon usage indices mentioned above were calculated using CodonW program [27].

## 3. Results

### 3.1. ENC-GC3s Plot

**Figure 1:**
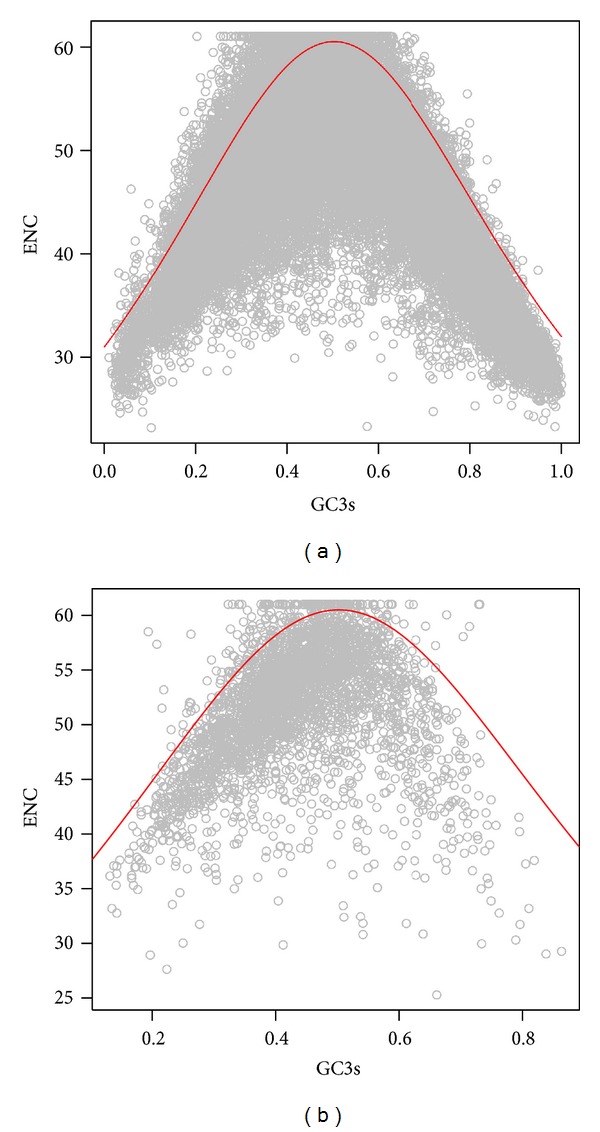
The relationship between ENC and GC3s for DNA (a) and RNA (b) virus genomes, respectively.

 As seen in Figure 1, the ENC-GC3s plot showed that most DNA or RNA virus genes lay on or slightly under the expected curve, indicating the extreme importance of mutational pressure for both groups of viruses. However, a great amount of points was laid under the curve as well for DNA (Figure 1(a)) and RNA (Figure 1(b)) viruses, suggesting that other factors, especially the influence of natural selection, were nontrivial. 

### 3.2. Influence of Gene Lengths and Protein Properties on GC Variation

**Figure 2:**
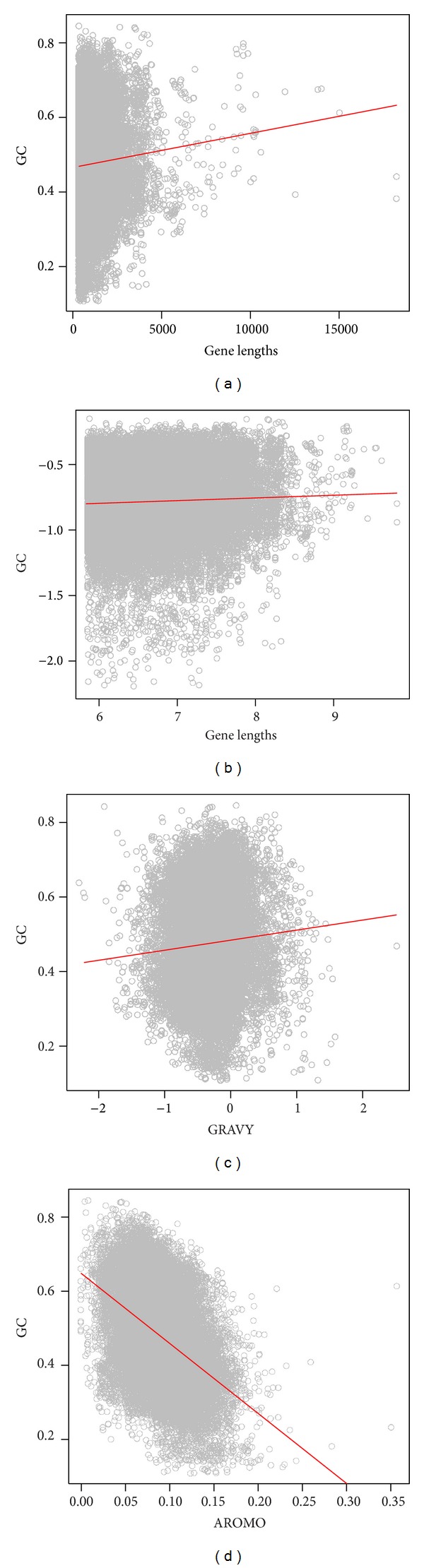
The relationships between GC content, gene length, and amino acid properties for DNA viruses. (a) GC-gene length relationship without transformation: GC = 9.14*E* −06 × gene  lengths + 0.466  (*R* ^2^ = 0.003, *P* < 0.0001); (b) GC-gene length relationship with log-transformation: log⁡_*e*_(GC) = 0.021 × log⁡_*e*_(gene  lengths) − 0.921  (*R* ^2^ = 0.002, *P* < 0.0001); (c) GC-hydrophobicity relationship: GC = −0.027 × GRAVY + 0.484  (*R* ^2^ = 0.005, *P* < 0.0001); (d) GC-aromaticity relationship: GC = −1.89 × AROMO + 0.648  (*R* ^2^ = 0.19, *P* < 0.001).

**Figure 3:**
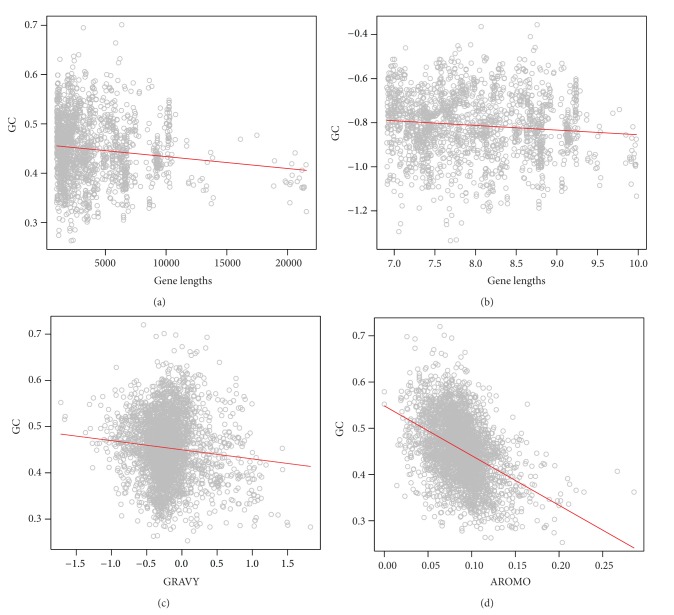
The relationships between GC content, gene length, and amino acid properties for RNA viruses. (a) GC-gene length relationship without transformation: GC = −3.19*E* − 06 × gene  lengths + 0.463  (*R* ^2^ = 0.021, *P* < 0.0001); (b) GC-gene length relationship with log-transformation: log⁡_*e*_(GC) = −0.0227 × log⁡_*e*_(gene  lengths) − 0.629  (*R* ^2^ = 0.021, *P* < 0.001); (c) GC-hydrophobicity relationship: GC = −0.009 × GRAVY + 0.452  (*R* ^2^ = 0.01, *P* < 0.05); (d) GC-aromaticity relationship: GC = −1.154 × AROMO + 0.555  (*R* ^2^ = 0.159, *P* < 0.001).

 Based on the present observation, there was a significant and positive correlation between GC content and gene lengths for DNA viruses (Figure 2(a)). The log-transformation further enhanced the positive trend (Figure 2(b)). However, for RNA viruses, the patterns became reverse: GC was significantly and negatively correlated with gene lengths for either original (Figure 3(a)) and log-transformed data points (Figure 3(b)). These results thus were incongruent with many previous studies working on a single of virus or a clade of viruses, which suggested that there were no clear trends between GC and gene lengths, for example, influenza viruses [28], polioviruses [10], parvoviridae [29], and so on. 

 Similar to the relationship between GC and gene lengths, there was an opposite relationship between GC and hydrophobicity for different types of viruses as well (Figures 2(c) and 3(c)). For DNA viruses (Figure 2(c)), the correlation between the two quantities was positive and significant, while for RNA viruses (Figure 3(c)), the correlation became positive and significant (Figure 3(c)). 

Finally, the relationship between GC and aromaticity was always negatively and significantly correlated for either DNA (Figure 2(d)) or RNA (Figure 3(d)) viruses. These significant correlations should suggest the signature of the influence of natural selection on codon usage patterns of viruses. 

### 3.3. Quantifying the Relative Ratio between Mutation and Selection Using Neutrality Plot on the Three Positions of Codons of Viruses

**Figure 4:**
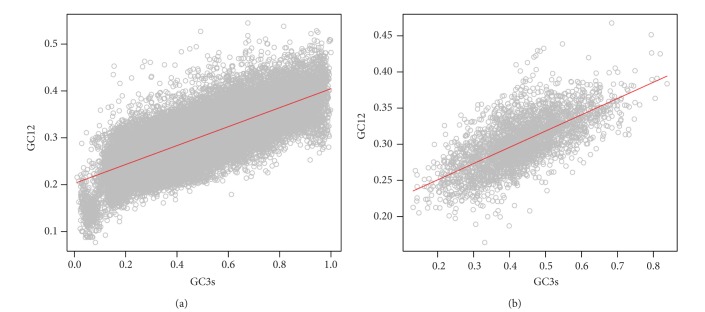
The relationship between GC12 and GC3s of DNA (a) and RNA (b) virus genomes. The fitted regression line has the formula as GC12 = 0.202 × GC3s + 0.203  (*R* ^2^ = 0.461, *P* < 0.0001) for DNA viruses and GC12 = 0.225 × GC3s + 0.206  (*R* ^2^ = 0.461, *P* < 0.0001) for RNA viruses, respectively.

 As shown in Figure 4(a), for DNA viruses, the correlation of GC3s and GC12 was best fitted by a linear function as GC12 = 0.202 × GC3s + 0.203(*R* ^2^ = 0.461, *P* < 0.0001) for DNA viruses. For RNA viruses, the linear regression model was as similar as GC12 = 0.225 × GC3s + 0.206(*R* ^2^ = 0.461, *P* < 0.0001) (Figure 4(b)). The slope of the GC12-GC3s regression line indicated the relative mutaion pressure functioned on the first and second codon positions in relation to that on the third codon position [30–32]. As seen, GC12 was influenced by mutation pressure and natural selection with a ratio being 0.202/0.798 = 0.253 for DNA viruses and 0.225/0.775 = 0.29 for RNA viruses correspondingly. These results indicated that the natural selection was more important on structuring the first and second codon positions and had similar influences for both groups of viruses. 

### 3.4. CA and CCA Analyses for Characterizing the Major Trends in Codon Usage Patterns of Viruses

**Figure 5:**
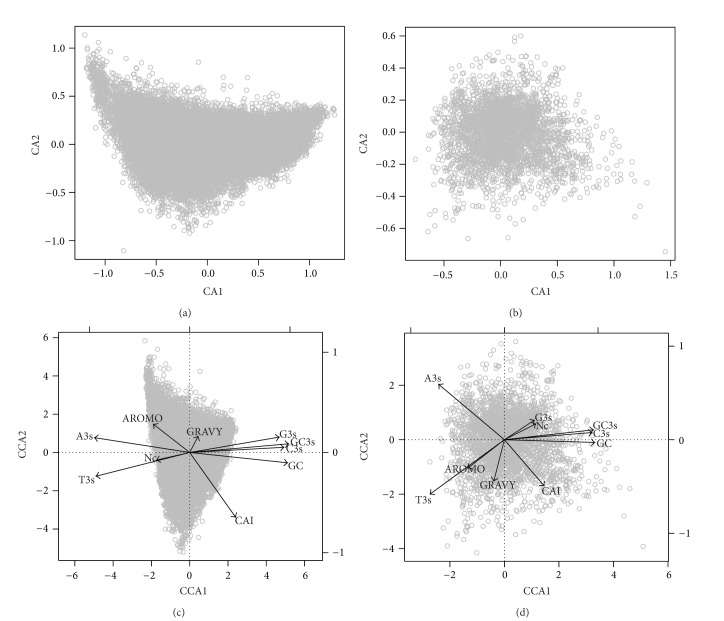
CA plots and CCA biplots for showing the major trends of codon usage patterns of the ORFs for DNA and RNA viruses. (a) CA plot for DNA viruses; (b) CA plot for RNA viruses; (c) CCA biplot for DNA viruses; (d) CCA biplot for RNA viruses.

For the ORFs of DNA viruses, the first (CA1) and second (CA2) axes of CA explained 34.5% and 5.6% of total variation in the codon usage patterns (Figure 5(a)). For RNA viruses, the first and second axes of CA explained 27.3% and 9.2% of the total variation, respectively, in synonymous codon usage patterns (Figure 5(b)). Thus, CA1 reflected the major trends for both DNA and RNA virus ORFs. 

For DNA viruses, CA1 was strongly correlated with GC3s (*r* = 0.99), followed by GC (*r* = 0.98) and A3s (*r* = −0.94), while CA2 was strongly related to CAI (*r* = −0.61), followed by AROMO (*r* = 0.23) and GRAVY (*r* = 0.17) (Table 1). The patterns for RNA viruses were similar for the first axis, which was most correlated with GC (*r* = 0.96), followed by GC3s (*r* = 0.94) and C3s (*r* = 0.91). However, the second axis CA2 was correlated with A3s (*r* = 0.59), T3s (*r* = −0.50), and CAI (*r* = −0.4) (Table 1). 

For DNA viruses, the first (CCA1) and second (CCA2) axes of CCA explained 68.3% and 8.8% of the total variation in synonymous codon usage patterns (Figure 5(c)). Being identical to the correlation results for CA as described above, CCA1 was strongly correlated with GC3s, GC, and A3s, while CCA2 was strongly related to CAI, AROMO, and T3s (Table 1). 

For RNA viruses, the first two axes explained 50.8% and 16.2% of total variation (Figure 5(d)). The most important variables correlated with CCA1 were identical as those for DNA viruses, while A3s, T3s, and CAI were the most important variables for CCA2 (Table 1). 

When comparing both CA and CCA results, it was consistently found that the following factors are repeatedly identified as most correlated ones for the principal axes for both DNA and RNA viruses: GC3s, GC, and A3s (Table 1). Thus, these variables should be of great importance to influence codon usage bias patterns for viruses.

### 3.5. Quantifying the Relative Importance of Mutation Pressure and Natural Selection in Overall Codon Usage Patterns of Viruses

**Figure 6:**
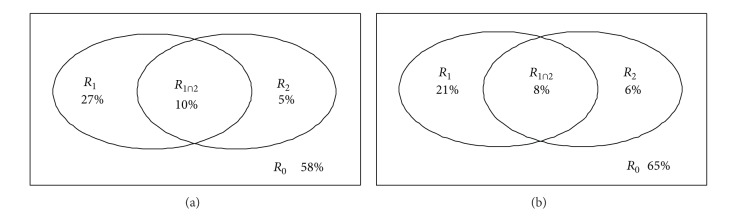
Variation partitioning of codon usage patterns attributed to mutation and selection for DNA (a) and RNA (b) viruses. The meaning of each part of the variation is interpreted as follows, *R*_1_: proportion of variation explained by mutation; *R*_2_: proportion of variation explained by selection; *R*_1∩2_: proportion of variation explained by the interaction of selection and mutation; *R*_0_: proportion of unexplained variation.

Based on the results of variation partitioning (Figure 6(a)), for DNA viruses, it was found that 27% of total variation could be attributed to mutational pressure, while only 5% of total variation of codon usage patterns attributed to selection pressure. The interaction between mutation and selection further explained 10% of total variation. Very similarly, for RNA viruses (Figure 6(b)), mutational pressure explained 21% of the total variation in codon usage patterns, while natural selection explained 6% of the total variation. The interaction of both mechanisms further explained 8% of the total variation. 

## 4. Discussion

### 4.1. The Relationship between GC Variation and Codon Usage Factors

Interestingly, it is found that the correlation between GC content and hydrophobicity and gene lengths is positive and significant for DNA viruses (Figures 2(a)–2(c)), while being negative and significant of RNA viruses (Figures 3(a)–3(c)). In contrast, the tendency between GC versus aromaticity is always negative (Figures 2(d) and 3(d)). The positive correlation between GC and hydrophobicity for RNA viruses is contradictory to some previous studies working on specific RNA virus species or clades, which argued that the correlation should be positive [33]. Moreover, it is still controversial whether there is a clear correlation for a specific virus or a clade of viruses. Some previous studies [3, 34] concluded that there was no clear relationship between these two quantities. 

I do not observe a congruent relationship between GC content and gene lengths for DNA and RNA viruses (Figures 2(a), 2(b), 3(a), and 3(b)). Based on some predictions, GC content should be correlated with gene length since selection should be stronger in longer genes, causing the directional change of GC content [35–38]. Indeed, GC has been thought to relate to gene lengths in prokaryotes, plants, nematodes, or insects [2, 22, 35, 36, 39], although the relationship among these taxa is still debatable [40]. On the basis of the results for DNA and RNA virus genomes at the present study, I argue that there is no consistent relationship between GC profiling and gene lengths for viruses. As shown in Figures 2(a) and 2(b), for DNA viruses, the relationship between GC and gene lengths is positive, implying the imprint of natural selection. However, for RNA viruses (Figures 3(a) and 3(b)), the relationship becomes negative, being opposite to the prediction of natural selection. This is not surprising, because RNA viruses are believed to have much higher mutation rates than DNA viruses [12, 28, 41]. At this perspective, viruses are different to other life forms from other kingdoms, in which natural selection plays differential roles to influence the stability of longer genes of DNA and RNA viruses. 

It is found that the prescreening and removal of short-length ORFs are very crucial to obtain accurate trends between GC and codon usage indices. For example, the negative relationship between GC and hydrophobicity may become obscured when more short-length ORFs (less than 350 bp) are included in the study for DNA viruses. The correlation will become nonsignificant (results not showed here).

### 4.2. Virus Genomes Are Profoundly Influenced by Mutation Pressure

 Based on the results of variation partitioning, the present study identifies that mutational pressure is the most prevailing mechanism driving the codon usage bias patterns for both DNA and RNA viruses (Figure 6), because it can explain 27% and 21% of total variation, respectively, in codon usage patterns of both groups of viruses. In contrast, the influence of natural selection is very minor, only explaining 5% and 6% of the total variation, respectively, for both groups of viruses. Previous studies on a single or a clade of viruses largely have confirmed the dominating influence of mutational pressure [6, 10, 42, 43], but many studies also mentioned the considerable importance of selection [29, 44]. Thus, through the present intergenomic analysis, I have a chance to quantify the relative importance of mutation versus selection on structuring codon usage patterns of viruses, and the similar conclusion is enforced: natural selection is not so important in comparison to mutational pressure in synonymous codon usage patterns of viruses. 

 Through correlation analysis between codon usage indices and major axes from CA and CCA analyses, the present study identifies that the three most important indices are GC, GC3s, and A3s. Thus, the present results are contradictory with a previous study [13] which showed that genomic nucleotide content was the most important factor predicting synonymous codon usage patterns in RNA viruses using randomization techniques. The difference raised may be partially due to the studied data size. In the previous study [13], only 29 RNA virus species were examined. This number is a very small number in comparison to the present study which works on 725 RNA viruses. Thus, the conclusion from the previous study [13] stating that GC content was a poor predictor of codon usage patterns of RNA viruses may be challenging if the authors can work on a large number of RNA viruses. 

 Finally, as implied by a large fraction of the unexplained variation (over 50%) for both groups of viruses, my quantification of mutational pressure and natural selection by utilizing the present ten codon usage variables might not be sufficient to quantify the relative importance of mutational pressure and natural selection. Other more important variables, especially those for characterizing natural selection (e.g., the frequency of usage of optimal codons [45]), might increase the explanatory power of natural selection on codon usage patterns of virus genomes.

### 4.3. Limitations of the Present Study

 I have to acknowledge that the recombination events happened at either gene or genome levels can influence the codon usage bias patterns to some extent, as evidenced by some previous studies [46–48]. Virus genomes have been broadly observed to process some degrees of homologous recombination [49–52]. As such, it would be a contribution when ones eliminate the influence of homologous recombination in the virus genomes before analyzing codon usage patterns to accurately disentangle the relative importance of mutation and selection. 

## Figures and Tables

**Table 1:** Correlation analysis of the first two axes of CA and explanatory variables for codon usage bias patterns of virus genomes. For each axis, the correlation coefficients for the top three important variables are marked in boldface.

Variables	DNA viruses				RNA viruses			
	CA1	CA2	CCA1	CCA2	CA1	CA2	CCA1	CCA2
T3s	−0.919	−0.158	−0.94	−**0.238**	−0.774	−**0.501**	−0.807	−**0.57**
C3s	0.929	−0.031	0.946	0.052	**0.912**	0.006	**0.945**	0.077
A3s	−**0.941**	0.165	−**0.956**	0.148	−0.687	**0.585**	−0.691	**0.622**
G3s	0.858	0.099	0.899	0.155	0.24	0.203	0.317	0.176
GC3s	**0.991**	0.022	**0.993**	0.088	**0.935**	0.048	**0.957**	0.088
GC	**0.98**	−0.13	**0.982**	−0.104	**0.959**	−0.094	**0.971**	−0.041
CAI	0.422	−**0.607**	0.463	−**0.652**	0.38	−**0.4**	0.424	−**0.488**
ENC	−0.261	−0.056	−0.335	−0.08	0.217	0.051	0.34	0.162
GRAVY	0.028	**0.172**	0.086	0.169	−0.058	−0.358	−0.158	−0.481
AROMO	−0.328	**0.23**	−0.368	**0.287**	−0.362	−0.25	−0.404	−0.306
